# Increasing the Receptor Tyrosine Kinase EphB2 Prevents Amyloid-β-induced Depletion of Cell Surface Glutamate Receptors by a Mechanism That Requires the PDZ-binding Motif of EphB2 and Neuronal Activity*

**DOI:** 10.1074/jbc.M115.666529

**Published:** 2015-11-20

**Authors:** Takashi Miyamoto, Daniel Kim, Joseph A. Knox, Erik Johnson, Lennart Mucke

**Affiliations:** From the ‡ Gladstone Institute of Neurological Disease, San Francisco, California 94158 and; § Department of Neurology, University of California, San Francisco, California 94158

**Keywords:** α-amino-3-hydroxy-5-methyl-4-isoxazolepropionic acid receptor (AMPA receptor, AMPAR), Alzheimer disease, amyloid-β (Aβ), N-methyl-D-aspartate receptor (NMDA receptor, NMDAR), neurodegeneration, prion, EphB2, PDZ, tau

## Abstract

Diverse lines of evidence suggest that amyloid-β (Aβ) peptides causally contribute to the pathogenesis of Alzheimer disease (AD), the most frequent neurodegenerative disorder. However, the mechanisms by which Aβ impairs neuronal functions remain to be fully elucidated. Previous studies showed that soluble Aβ oligomers interfere with synaptic functions by depleting NMDA-type glutamate receptors (NMDARs) from the neuronal surface and that overexpression of the receptor tyrosine kinase EphB2 can counteract this process. Through pharmacological treatments and biochemical analyses of primary neuronal cultures expressing wild-type or mutant forms of EphB2, we demonstrate that this protective effect of EphB2 depends on its PDZ-binding motif and the presence of neuronal activity but not on its kinase activity. We further present evidence that the protective effect of EphB2 may be mediated by the AMPA-type glutamate receptor subunit GluA2, which can become associated with the PDZ-binding motif of EphB2 through PDZ domain-containing proteins and can promote the retention of NMDARs in the membrane. In addition, we show that the Aβ-induced depletion of surface NMDARs does not depend on several factors that have been implicated in the pathogenesis of Aβ-induced neuronal dysfunction, including aberrant neuronal activity, tau, prion protein (PrP^C^), and EphB2 itself. Thus, although EphB2 does not appear to be directly involved in the Aβ-induced depletion of NMDARs, increasing its expression may counteract this pathogenic process through a neuronal activity- and PDZ-dependent regulation of AMPA-type glutamate receptors.

## Introduction

Alzheimer disease (AD)^3^ is the most common neurodegenerative disorder, affecting millions of elderly people worldwide (1). Disease-modifying treatments are urgently needed because the number of AD patients is rising as many populations across the world live longer, reaching ages at which the risk of AD is high. It is likely that the development of such treatments will require a deeper understanding of AD pathogenesis, which appears to be multifactorial (2). Diverse lines of evidence suggest that amyloid-β (Aβ) oligomers causally contribute to cognitive impairments associated with AD at least partly by altering synaptic activity (2–6). Synaptic activity and cognition critically depend on the function of NMDA-type glutamate receptors (NMDARs) and AMPA-type glutamate receptors (AMPARs), both of which appear to be affected by Aβ oligomers. Indeed, Aβ oligomers deplete and dysregulate NMDARs (7–12) and AMPARs (9, 13, 14). However, the precise molecular paths that lead from Aβ oligomers to alterations in these glutamate receptors remain to be elucidated.

Several factors, including tau, aberrant network activity, and prion protein (PrP^C^), may mediate or enable Aβ-induced neuronal dysfunctions. We and others showed that tau reduction prevents synaptic, network, and behavioral abnormalities in multiple human amyloid precursor protein (hAPP) transgenic mouse lines that have pathologically elevated levels of Aβ oligomers in brain (15–18). Several studies suggest that aberrant excitatory network activity, which tau reduction can block (15–17), contributes to synaptic and cognitive deficits in hAPP mice and in patients with early stages of AD (19–23). PrP^C^ has also been reported to be required for Aβ-induced neuronal and cognitive impairments and has been proposed to mediate Aβ-induced depletion of NMDARs (24–27), although several of these findings could not be reproduced by other groups (28–30).

Another interesting molecule that may connect Aβ oligomers to glutamate receptors is the receptor tyrosine kinase EphB2 (12, 31, 32), which regulates the trafficking and function of NMDARs (33). Aβ oligomers directly bind to EphB2 (32) and cause its degradation (32). Neuronal overexpression of hAPP/Aβ in transgenic mice or neuronal knockdown of EphB2 in wild-type mice each impaired long term potentiation and NMDAR-mediated currents (32). Furthermore, normalizing EphB2 levels in the dentate gyrus of hAPP mice reversed some of their synaptic and cognitive impairments (32). Taken together, these findings motivated us to investigate the relationship among Aβ oligomers, EphB2, and glutamate receptors in greater detail and to determine the extent to which their interactions depend on tau, PrP^C^, and neuronal activity.

## Experimental Procedures

### 

#### 

##### Mice

*Mapt* knock-out (tau-deficient) mice (34) were kindly provided by Dr. Hana Dawson (Duke University) and were maintained on a C57Bl/6J background. *Prnp* knock-out (PrP^C^-deficient) mice (B6.129S7-Prnp^tm1Cwe^/Orl) (35) were acquired from the European Mutant Mouse Archive and had been crossed onto the C57Bl/6J strain for >10 generations. *Ephb2* knock-out (EphB2-deficient) mice (36) were kindly provided by Dr. Mark Henkemeyer (University of Texas Southwestern). They were obtained on a CD1 background and rederived using C57Bl/6J females.

##### Recombinant Aβ Oligomers

Unless indicated otherwise, the Aβ oligomers we used to treat primary neuronal cultures were prepared from recombinant Aβ peptides, and statements made about Aβ oligomers refer to this type of Aβ assembly. In brief, hydroxyfluroisopropanol-treated recombinant Aβ peptides (β-amyloid(1–42), ultra pure, hydroxyfluroisopropanol from rPeptide, catalogue number A1163, 0.5 mg, primary lot number 9131142H) were first dissolved in 22 μl of DMSO (at ∼2.5 mm Aβ(1–42) peptides, monomer equivalent) and then further diluted with 978 μl of ice-cold Neurobasal A medium to generate a ∼50 μm Aβ(1–42) solution. The Aβ(1–42) solution was incubated at 4 °C for 24 h to oligomerize Aβ(1–42) peptides. Vehicle solution was prepared by following the same protocol except for omitting addition of Aβ(1–42) peptides. On the day of the experiment, the concentration of Aβ(1–42) peptides was determined by bicinchoninic acid (BCA) assay (Thermo Scientific, 23225), and vehicle control or oligomerized Aβ(1–42) peptides (final concentration of 2 μm monomer equivalent) were applied to primary neuronal cultures.

To characterize Aβ oligomers by atomic force microscopy (AFM), Aβ(1–42) peptides dissolved in 22 μl of DMSO were diluted with 978 μl of ice-cold Dulbecco's phosphate-buffered saline without calcium or magnesium (DPBS-no Ca^2+^/Mg^2+^; Life Technologies, 14190-144) because Neurobasal medium contains factors that interfere with AFM analysis. When added to the medium of neuronal cultures, Aβ oligomers prepared in DPBS-no Ca^2+^/Mg^2+^ depleted cell surface GluN1 and EphB2 after 2 and 48 h, respectively (data not shown), confirming their bioactivity.

##### Size Exclusion Chromatography (SEC) and Aβ ELISA

We collected culture medium containing Aβ oligomers 2 and 48 h after treating cultured neurons and injected it onto a Superdex 75 (10/300GL) column (GE Healthcare) calibrated using a gel filtration standard kit (Bio-Rad, 151-1901). Samples were eluted with 1 column volume of phosphate-buffered saline (PBS) at a flow rate of 0.8 ml/min into 1-ml SEC fractions. Eluted fractions were further diluted 1:1000, 1:2000, and 1:4000 for Aβ(1–42) ELISA, which was performed as described (37).

##### Primary Hippocampal Mouse Neurons

Unless indicated otherwise, experiments were carried out on primary hippocampal mouse neurons. Hippocampi of newborn mouse pups (P0-P1) were dissected in ice-cold Earle's balanced salt solution without CaCl_2_, MgSO_4_, and phenol red (Life Technologies, 14155). Dissected hippocampi were digested with papain (Worthington, LK003176; ∼1 unit per hippocampus) in Earle's balanced salt solution at 37 °C for 15 min and then triturated in a disposable plastic tube in low ovomucoid solution containing 1.5 mg/ml BSA (Sigma-Aldrich, A7030-10G), 1.5 mg/ml trypsin inhibitor (Sigma-Aldrich, T9253-5G), and 66.7 units/ml DNase I (Sigma-Aldrich, D5025) in DPBS (Life Technologies, 14040-182). After removing debris with a 70-μm nylon strainer (BD Biosciences, 352350), neurons were spun at 1000 rpm for 5 min. Cell pellets were gently dissociated in Neurobasal A medium supplemented with 1× B27 (Life Technologies, 17504-044), 2.4 mm l-glutamine (Life Technologies, 25030-081), and 100 units/ml penicillin/streptomycin (Life Technologies, 15410-122). They were then plated on poly-d-lysine-coated 12-well plates at a density of 500,000 neurons/well for Western blot analyses and 1,000,000 neurons/coverslip for proximity ligation assay. Half of the medium was replaced with new medium every week, and neurons were used for experiments at DIV 10–14.

##### Biotinylation and Isolation of Cell Surface Proteins

Cell surface proteins were biotinylated and isolated using a cell surface protein isolation kit (Thermo Scientific, 89881). After various treatments, primary hippocampal mouse neurons (DIV 10–14) were washed once with ice-cold PBS and then incubated with sulfo-NHS-SS-biotin (sulfosuccinimidyl-2-(biotinamido)-ethyl-1,3-dithiopropionate; 0.25 mg/ml in ice-cold PBS) for 30 min at 4 °C. After quenching the biotinylation reaction, neurons were washed twice with ice-cold Tris-buffered saline (TBS) and lysed in Pierce IP Lysis Buffer (Thermo Scientific, 87788) with Halt protease and phosphatase inhibitor mixture (Thermo Scientific, 78440). Lysates were then sonicated on ice using five 1-s bursts and centrifuged at 1000 rpm for 5 min at 4 °C followed by determination of protein concentration by BCA protein assay. To isolate biotinylated surface proteins, 30 μg of biotinylated total protein was incubated with NeutrAvidin gel slurry (25 μl) at room temperature for 1 h followed by two washes with TBS and two additional washes with Pierce IP Lysis Buffer. Isolated biotinylated proteins were then solubilized in loading buffer for Western blot analyses.

##### Western Blot Analysis

Equal amounts of total protein (15 μg per lane), or entire eluate from 25 μl of NeutrAvidin gel incubated with 30 μg of biotinylated protein lysate, in 1× NuPAGE lithium dodecyl sulfate sample buffer (Life Technologies, NP0007) and 1× Sample Reducing Agent (Life Technologies, NP0009) were loaded per gel lane. Protein samples were electrophoresed on NuPAGE Novex 4–12% Bis-Tris Midi protein gels (Life Technologies, WT1403A) in 1× NuPAGE MOPS SDS running buffer (Life Technologies, NP0001-02) at 200 V for 1 h at room temperature. Gels were transferred to nitrocellulose membranes using an iBlot gel transfer device (Life Technologies). Membranes were blocked with Odyssey blocking buffer (LI-COR Biosciences, 927-40000) for 1 h at room temperature and then incubated with the primary antibodies listed in Table 1 overnight at 4 °C. Membranes were washed with TBS containing 0.05% Tween 20 (TBST) four times for 5 min at room temperature and then incubated with matching secondary antibodies conjugated to IRDye (Li-COR Biosciences; 0.1 μg/ml) for 1 h at room temperature followed by washes in TBST (4 × 5 min). Protein bands were visualized using an Odyssey CLx Infrared Imaging System (LI-COR Biosciences) and quantified with Image Studio software (LI-COR Biosciences).

**TABLE 1:** **Primary antibodies used for Western blotting**

Antibody	Source	Final concentration
βIII-tubulin	Sigma-Aldrich, T5076-200UL	0.1 μg/ml
EphA2	Millipore, 05-480	0.2 μg/ml
EphB2	R&D Systems, AF467	0.1 μg/ml
ERK (phosphorylated)	Cell Signaling Technology, 9101	1:2000
ERK (total)	Cell Signaling Technology, 9102	1:1000
FLAG	Sigma-Aldrich, F1804	2.0 μg/ml
GluA2	NeuroMab, clone L21/32	0.1 μg/ml
GluN1	Millipore, AB9864R	0.1 μg/ml

**FIGURE 1.**
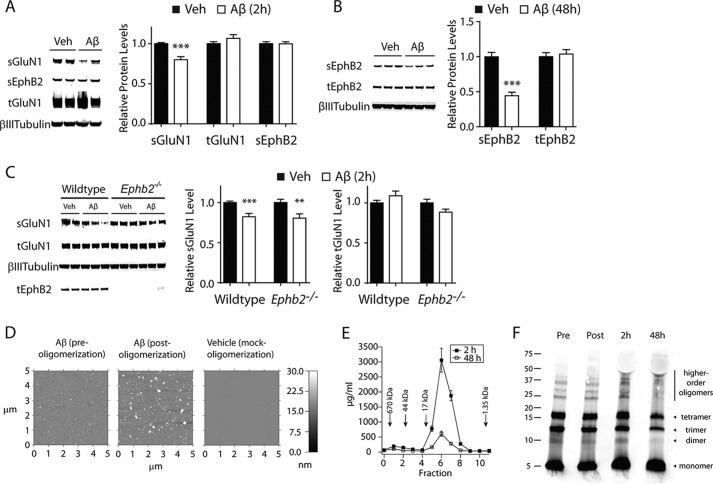
**Aβ-induced depletion of surface GluN1 in primary neuronal cultures precedes and does not depend on depletion of surface EphB2.** *A–C*, cultures of primary hippocampal neurons (DIV 10–14) from wild-type (*A–C*) and *Ephb2*^−/−^ (*C*) mice were treated with Aβ oligomers (2 μm) or vehicle (*Veh*; 0.08% DMSO) and then analyzed for surface (*s*) and total (*t*) levels of GluN1 and EphB2 by Western blotting. βIII-tubulin was used as a loading control for total protein levels. Representative Western blots are shown on the *left*, and quantitations of Western blot signals are shown on the *right*. Protein levels in vehicle-treated wild-type neurons were arbitrarily defined as 1.0. *A*, sGluN1, but not sEphB2, levels were reduced after 2 h of Aβ exposure. *n* = 26–30 wells per condition from eight independent experiments. *B*, sEphB2 levels were reduced after 48 h of Aβ exposure. *n* = 12 wells per condition from four independent experiments. *C*, 2 h of Aβ exposure reduced sGluN1 levels in wild-type and EphB2-deficient neurons. *n* = 23–24 wells per condition from four to five independent experiments. *D–F*, Aβ oligomers were characterized by AFM (*D*), ELISA on SEC fractions (*E*), and Western blot analysis (*F*). *D*, AFM images of Aβ(1–42) peptides before (*left*) and after (*middle*) oligomerization and of a vehicle control (*right*). Note the globular bright structures representing putative Aβ oligomers in the *middle panel. E*, medium removed from neuronal cultures 2 (*black*) or 48 (*white*) h after addition of Aβ oligomers were fractionated by SEC, and Aβ(1–42) concentrations in each fraction were determined by ELISA. *Arrows* indicate the relation of fractions to the molecular weight of eluted peptide assemblies. *F*, preparations of Aβ(1–42) peptides before (*Pre*) and after (*Post*) oligomerization and medium removed from neuronal cultures 2 or 48 h after addition of Aβ oligomers were analyzed by Western blotting using a combination of anti-Aβ antibodies 82E1 and 6E10. Molecular mass markers (kDa) are indicated on the *left*. **, *p* < 0.01; ***, *p* < 0.001 *versus* vehicle (unpaired *t* test with Welch's correction). *Bars* and *error bars* represent means and S.E., respectively.

For Western blot analysis of Aβ oligomers (Fig. 1*F*), PBS or Neurobasal medium containing 0.5 μg of Aβ in 1× NuPAGE lithium dodecyl sulfate sample buffer was loaded per gel lane. Aβ samples were electrophoresed on a 10–20% Criterion Tris-Tricine gel (Bio-Rad, 3450068) in 2× XT MES Running Buffer (Bio-Rad, 1610789) at 150 V for 3 h at 4 °C. Gels were transferred to nitrocellulose membranes in 1× NuPAGE transfer buffer (Life Technologies, NP0006-1) containing 10% methanol at 0.3 A for 2 h at 4 °C. Membranes were then microwaved for 5 min in PBS for antigen retrieval, blocked in 5% BSA (Sigma-Aldrich, A3803-100G) in TBS overnight at 4 °C, and incubated with a combination of two anti-Aβ antibodies (82E1 from IBL America (10323) at 1:1000 dilution and 6E10 from Covance (SIG-39320) at 1:2000 dilution) in TBS containing 5% BSA for 2 h at room temperature. Membranes were then washed with TBST four times for 5 min at room temperature and incubated with goat anti-mouse secondary antibodies conjugated to IRDye (0.1 μg/ml) for 1 h at room temperature followed by washes in TBST (4 × 5 min). Protein bands were visualized using an Odyssey CLx Infrared Imaging System and quantified with Image Studio software.

##### Generation of EphB2 Deletion Mutants

Deletion mutants of EphB2 with two FLAG tags inserted at the N-terminal side of the ligand-binding domain were generated using a QuikChange II XL site-directed mutagenesis kit (Agilent Technologies, 200522). FLAG-tagged EphB2 in pFUW plasmid (32) was used as a template in combination with the set of primers listed in Table 2. After mutagenesis, full sequences of deletion mutants were verified. Plasmids carrying the desired mutations were used to transform the Stbl3 *Escherichia coli* strain (Life Technologies, C7373-03) for maintenance.

**TABLE 2:** **Primers used to generate deletion mutants of FLAG-tagged EphB2** s, sense; as, antisense.

Primer ID	Sequence
K661R_as	5′-atcctgacttgagggtcctgatggctacaaagatc-3′
K661R_s	5′-gatctttgtagccatcaggaccctcaagtcaggat-3′
ΔFN_as	5′-actcggcttctgtcatgatggttgtgcaaggc-3′
ΔFN_s	5′-gccttgcacaaccatcatgacagaagccgagt-3′
ΔLB_as	5′-gaagatggcaccattcacggcggctagcag-3′
ΔLB_s	5′-ctgctagccgccgtgaatggtgccatcttc-3′
ΔPDZ_as	5′-gatgaaccagatccagtgacggaccggttacc-3′
ΔPDZ_s	5′-ggtaaccggtccgtcactggatctggttcatc-3′
ΔSAM_as	5′-gactggatctggttcatgtagtccggtatcgtgc-3′
ΔSAM_s	5′-gcacgataccggactacatgaaccagatccagtc-3′

##### Production and Purification of Lentiviral Particles

Lentiviral particles were generated by co-transfecting the transfer vector (pFUW with wild-type or mutant EphB2 cDNA insertion), the HIV-1 packaging vector (Delta8.9), and the VSVG envelope glycoprotein expression vector (pVSVG) into HEK293T cells. Confluent HEK293T cells were transfected with three vectors (22.5 μg of pFUW, 16.9 μg of Delta8.9, and 11.25 μg of pVSVG per 15-cm Petri dish) using CalPhos transfection reagent (Clontech, 631312) according to the manufacturer's instruction. Medium containing lentiviral particles was collected 48 h after transfection and filtered through a 0.22-μm cellulose acetate filter (Corning Inc., 431154). Lentiviral particles in the medium were then concentrated by serial ultracentrifugation: 21,000 rpm for 2 h at 4 °C in a Beckman SW28 and then 25,000 rpm for 2 h at 4 °C in a Beckman SW55 with a sucrose cushion (2 ml of 20% sucrose in Hanks' balanced salt solution (Life Technologies, 14170) at the bottom of the SW55 tubes). Final pellets were dissolved in Hanks' balanced salt solution, aliquoted, and stored at −80 °C until use. Lentiviral titers were determined with a p24 ELISA by Dr. David Chung (University of California, San Francisco). Primary cultured hippocampal neurons were transduced with lentiviral particles encoding EphB2 at 0.02 pg of p24/neuron. Lentiviral vectors encoding shRNA against GluN1 (sh-GluN1) or EphA2 were purchased from Sigma-Aldrich or GeneCopoeia, Inc., respectively. Primary cultured hippocampal neurons were transduced with these lentiviral particles at a multiplicity of infection of 2. We first compared the efficacies of five sh-GluN1 lentiviruses (Mission lentiviral transduction particles; clone IDs TRCN0000233326, TRCN0000233327, TRCN0000233328, TRCN0000233329, and TRCN0000257394) and selected two equally effective sh-GluN1 constructs (TRCN0000233326 and TRCN0000257394) for subsequent experiments. Because the results obtained with these two lentiviruses were similar (data not shown), they were combined for statistical analysis and data presentation.

##### Proximity Ligation Assay (PLA)

At DIV 7, primary hippocampal neuronal cultures from wild-type P0-P1 pups (10^6^ neurons plated on 12-mm poly-d-lysine/laminin-coated glass coverslips (BD Biosciences, 354087)) were transfected with empty pFUW plasmid or plasmids encoding EphB2^WT^ or EphB2^ΔPDZ^. GFP-encoding plasmid was co-transfected to visualize transfected neurons. To prepare the transfection mixture, pairs of plasmids (1.0 μg/coverslip each) were dissolved in 50 μl of Opti-MEM (Life Technologies, 31985-062), mixed with 50 μl of Opti-MEM containing 1.35 μl of Lipofectamine 2000 (Life Technologies, 11668-027), and incubated for 20 min at room temperature. The transfection mixture was added to neuronal cultures that had been incubated in Neurobasal A medium containing kynurenic acid (1 mm; Sigma-Aldrich, K3375-5G) and GlutaMAX (0.5 mm; Life Technologies, 35050-061) at 37 °C with 5% CO_2_ for 30–60 min. Neuronal cultures were incubated in the same transfection mixture for another 30 min at 37 °C with 5% CO_2_. Cultures were then washed with prewarmed PBS once and placed back into presaved conditioned medium. PLA was performed according to the protocol of Duolink In Situ/Fluorescence (Sigma-Aldrich) 1 week after transfection (at DIV 14). Neurons were fixed with 4% paraformaldehyde, 4% sucrose in 1× PBS for 15 min at room temperature, washed with 1× PBS (3 × 5 min), permeabilized with 0.2% Triton X-100 in 1× PBS for 5 min, and then blocked with 5% normal donkey serum, 1× PBS for 30 min at room temperature. They were then incubated with primary antibodies (goat anti-EphB2 at 0.4 μg/ml and mouse anti-GluA2 at 0.4 μg/ml) in 1% normal donkey serum, 1× PBS at 4 °C overnight. After washing with 1× PBS (3 × 5 min), neurons were incubated with two PLA probes (Duolink In Situ PLA Probes Anti-Goat PLUS DUO92003 and Anti-Mouse MINUS DUO92004, Sigma-Aldrich) in 1% normal donkey serum, 1× PBS for 1 h at 37 °C followed by a wash with 1× Wash Buffer A (2 × 5 min; Sigma-Aldrich, DUO82049-4L) and incubation in ligation solution (Sigma-Aldrich, Duolink In Situ Detection Reagents Red, DUO92008) for 1 h at 37 °C to ligate complimentary PLA probes that were in close (<40-nm) proximity (38). After ligation, neurons were washed with 1× Wash Buffer A (2 × 2 min) and incubated in the amplification solution (Duolink In Situ Detection Reagents Red) for 100 min at 37 °C to fluorescently label the ligated PLA probes. Neurons were then serially washed in 1× Wash Buffer B (2 × 10 min; Sigma-Aldrich, DUO82049-4L), 0.01× Wash Buffer B (1 × 1 min), and 1× PBS (1 × 5 min) followed by incubation with secondary antibodies (Alexa Fluor 647 donkey anti-goat, A21447 or Alexa Fluor 647 donkey anti-mouse, A-31571; Life Technologies) in 1% normal donkey serum, 1× PBS for 1 h at room temperature. Finally, neurons were washed with 1× PBS (3 × 5 min) and mounted in Duolink In Situ Mounting Medium with DAPI (Sigma-Aldrich, DUO82040). Fluorescence images were obtained with an epifluorescence microscope (Nikon Ti-E microscope, Nikon Imaging Center at University of California, San Francisco). Fluorescence intensity of the PLA signal in cell bodies (excluding the nucleus), EphB2 or GluA2 immunofluorescence, and GFP fluorescence were quantified using NIS Elements software (Nikon).

##### Transient Transfections

HEK293T cells were transfected with plasmids using Lipofectamine 2000 according to the manufacturer's instructions. To prepare the transfection mixture, plasmids encoding EphB2^WT^, EphB2^K661R^, or GluN2B (2.0 μg/well each) were dissolved in 100 μl of Opti-MEM, mixed with 100 μl of Opti-MEM containing 3 μl of Lipofectamine 2000, and incubated for 20 min at room temperature. The transfection mixture was then added to semiconfluent HEK293T cells plated on 12-well plates in DMEM high glucose (Life Technologies, 11965-092) supplemented with 2.4 mm l-glutamine, and 100 units/ml penicillin/streptomycin. Cells were used for experiments 24 h later.

##### Preclustering of Recombinant Fc-EphrinB2

Before addition to HEK293T cells expressing EphB2, recombinant Fc-EphrinB2 (5 μg/ml; R&D Systems, 496-EB-200), a fusion protein of mouse EphrinB2 and human IgG1 (39), was preclustered with goat anti-human antibody (0.5 μg/ml; Jackson ImmunoResearch Laboratories, 109-001-008) in serum-free DMEM for 1 h on a shaker at room temperature. Preclustered Fc-EphrinB2 was diluted 1:10 and applied to HEK293T cells for 1 h at 37 °C.

##### Other Compounds Used

Tetrodotoxin (TTX) (catalogue number 1078), dl-*2-amino-5-phosphonovaleric acid* (0105), cyclosporin A (1101), FK506 (3631), and α-bungarotoxin (2133) were purchased from Tocris Bioscience and used as described in the text.

##### Statistical Analysis

Experimenters were blinded with respect to the genotype and treatment of cell cultures. Biological units were randomized during assays, sampling, and analyses. Statistical analyses were performed with Prism (version 6, GraphPad) and R (R Development Core Team). Individual culture wells (Western blot data) or individual transfected neurons (PLA data) were treated as independent biological units (*n*). Differences between genotypes and treatments were assessed, as appropriate, by unpaired Student's *t* test with Welch's correction or by one-way or two-way ANOVA with Bonferroni multiple comparison post hoc test. The null hypothesis was rejected at *p* < 0.05. In all figures, quantitative data are presented as means ± S.E.

## Results

### 

#### 

##### Aβ Oligomers Deplete NMDARs and EphB2 from the Neuronal Surface through Independent Mechanisms

Two-hour treatment of primary hippocampal neuronal cultures with recombinant Aβ oligomers (2 μm; monomer equivalent) reduced levels of surface GluN1 (sGluN1) (Fig. 1*A*), an obligatory subunit of NMDARs, consistent with previous reports (7, 10). At this time point, levels of surface EphB2 (sEphB2) were still unchanged (Fig. 1*A*). Reductions of sEphB2 levels were observed after 48 h of Aβ treatment (Fig. 1*B*), consistent with previous findings (12, 32). Notably, 2-h treatment with Aβ oligomers reduced sGluN1 levels also in primary hippocampal neurons from mice lacking EphB2 (Fig. 1*C*). Thus, at least in these mouse cultures, Aβ-induced depletion of sGluN1 does not depend on EphB2 or alterations in its surface levels.

We confirmed the oligomeric nature of the recombinant Aβ preparations used in these experiments by atomic force microscopy (Fig. 1*D*). We also removed medium from neuronal cultures 2 and 48 h after addition of Aβ oligomers and characterized the Aβ species they contained by Western blot analysis as well as by SEC and ELISA (Fig. 1, *E* and *F*). The medium contained higher levels of Aβ oligomers at 2 h than at 48 h after addition of Aβ oligomers (Fig. 1, *E* and *F*), possibly because, with time, more and more Aβ oligomers bind to neuronal surface membranes (40) and are sequestered by the formation of Aβ fibrils. Western blot analysis revealed putative Aβ oligomers even in preparations of freshly solubilized Aβ peptides (Fig. 1*F*), likely due in part to the fact that SDS promotes the oligomerization of Aβ(1–42) in gels (41).

**FIGURE 2.**
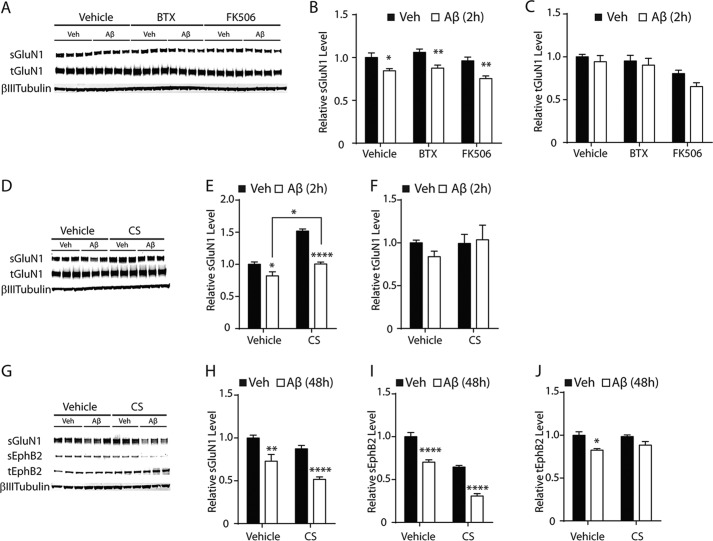
**Aβ-induced depletion of sGluN1 occurs in the presence of α-bungarotoxin, FK506, or cyclosporin A.** *A–C*, cultures of primary hippocampal neurons (DIV 12–14) from wild-type mice were treated with Aβ oligomers (2 μm) or vehicle (*Veh*; 0.08% DMSO) for 2 h followed by Western blot analysis of sGluN1 and tGluN1. Some cultures were treated with BTX (10 μm) or FK506 (50 μm) from 30 min before until the end of exposure to Aβ or vehicle. *A*, representative Western blots. *B* and *C*, quantitation of sGluN1 (*B*) and tGluN1 (*C*) levels. *n* = 8 wells per condition from two independent experiments. *D--I*, cultures of primary hippocampal neurons (DIV 12) from wild-type mice were treated with Aβ oligomers (2 μm) or vehicle (*Veh*; 0.08% DMSO) for 2 (*D--F*) or 48 (*G–J*) h followed by Western blot analysis of surface (*s*) and total (*t*) levels of GluN1 and EphB2. Some cultures were treated with cyclosporin A (*CS*; 20 μm) from 30 min before until the end of exposure to Aβ or vehicle. *D* and *G*, representative Western blot. *E* and *F*, quantitations of sGluN1 (*E*) and tGluN1 (*F*) levels. *n* = 6 wells per condition from one experiment. *H–J*, quantitations of sGluN1 (*H*), sEphB2 (*I*), and tEphB2 (*J*) levels. *n* = 6 wells per condition from one experiment. Two-way ANOVA revealed a significant interaction between Aβ oligomers and compounds in *E* (*p* < 0.001); a significant effect of Aβ oligomers in *B*, *E*, *H*, and *I* (*p* < 0.0001); and significant effects of compounds in *B* (*p* < 0.05), *E* (*p* < 0.0001), *H* (*p* < 0.0001), and *I* (*p* < 0.0001). There were no significant interactions between Aβ oligomers and compounds in *B*, *H*, and *I*. *, *p* < 0.05; **, *p* < 0.01; ****, *p* < 0.0001 *versus* vehicle or as indicated by *bracket* in *E* (Bonferroni test). *Bars* and *error bars* represent means and S.E., respectively.

In light of the known interactions between NMDARs and EphB2 (33), we next examined whether depletion of NMDARs may underlie the subsequent depletion of sEphB2. We first examined whether known inhibitors of Aβ-induced sGluN1 depletion block the subsequent depletion of sEphB2. α-Bungarotoxin (BTX), a blocker of α-7 nicotinic acetylcholine receptors (α-7), has been reported to partially block Aβ-induced sGluN1 depletion (7). Two antagonists of the protein phosphatase calcineurin, FK506 and cyclosporin A, were also reported to block Aβ-induced sGluN1 depletion (7) and synaptic depression (9). However, treatment of neuronal cultures with BTX (10 μm) or FK506 (50 μm) did not prevent Aβ-induced depletion of sGluN1 (Fig. 2, *A–C*). Cyclosporin A (20 μm) increased sGluN1 levels and counteracted Aβ-induced sGluN1 depletion at 2 h (Fig. 2, *D–F*). However, 48-h treatment with cyclosporin A actually exacerbated the Aβ-induced depletion of sGluN1 and sEphB2 (Fig. 2, *G–J*).

**FIGURE 3.**
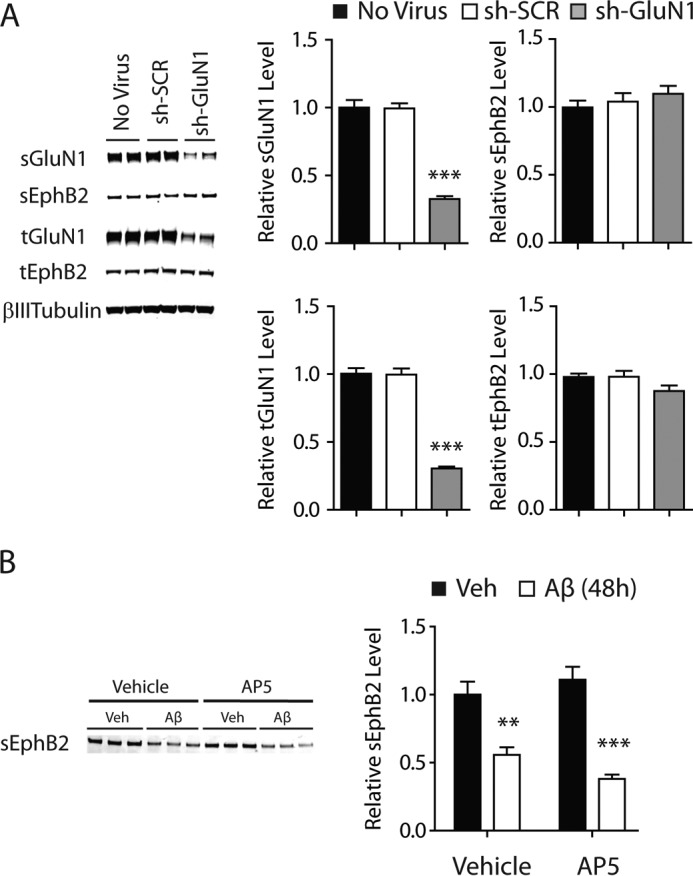
**Depleting or blocking NMDARs does not affect EphB2 levels in cultured neurons.** *A*, cultures of primary hippocampal neurons (DIV 2) from wild-type mice were not transduced (*No Virus*) or transduced with lentivirus (multiplicity of infection of 2) encoding scrambled shRNA (sh-SCR) or shRNA against GluN1 (sh-GluN1) followed by Western blot analysis of GluN1 and EphB2 levels at DIV 14. Cultures were not treated with Aβ. *n* = 10–26 wells per condition from two independent experiments. *B*, cultures of primary hippocampal neurons (DIV 10–14) from wild-type mice were treated for 48 h with Aβ oligomers (2 μm) or vehicle (*Veh*; 0.08% DMSO) in the presence of the NMDAR antagonist AP5 (100 μm) or vehicle (0.1% water) followed by Western blot analysis of sEphB2 levels. Representative Western blots are shown on the *left*, and quantitations of Western blot signals are shown on the *right. n* = 9 wells per condition from two independent experiments. Two-way ANOVA revealed no significant interaction between the effects of Aβ and AP5 on sEphB2 levels. **, *p* < 0.01; ***, *p* < 0.001 *versus* sh-SCR (*A*) or vehicle (*B*) by Bonferroni test. *Bars* and *error bars* represent means and S.E., respectively.

To evaluate the dependence of sEphB2 levels on sGluN1 levels more directly, we transduced neurons with lentiviral vectors expressing an anti-GluN1 short hairpin RNA (sh-GluN1) or a scrambled shRNA (sh-SCR). Transducing neurons with lentivirus encoding sh-GluN1 depleted both total and surface levels of GluN1 more markedly and for much longer (DIV 2–14) than the 2-h Aβ exposure, but it had no effect on levels of total or surface EphB2 (Fig. 3*A* and data not shown). These results suggest that depletion of sGluN1 is not sufficient to lower sEphB2 levels and that Aβ oligomers may deplete sGluN1 and sEphB2 through parallel mechanisms.

Because NMDAR activity is required for Aβ oligomers to impair neuronal functions (42), we also examined whether blocking NMDAR activity attenuates Aβ-induced sEphB2 depletion. Treatment of neuronal cultures with the NMDAR antagonist AP5 (100 μm) had no effect on Aβ-induced sEphB2 depletion (Fig. 3*B*), suggesting that the Aβ-induced depletion of sEphB2 does not require NMDAR activity.

##### Aβ-induced Depletion of sGluN1 and sEphB2 Does Not Require Tau, PrP^C^, or Neuronal Activity

**FIGURE 4.**
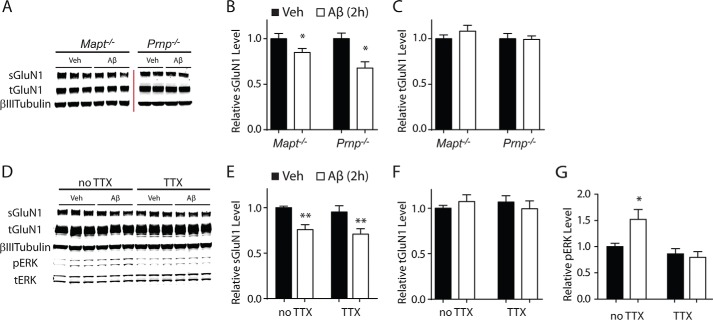
**Aβ-induced depletion of sGluN1 occurs in the absence of tau, PrP^C^, or neuronal activity.** *A–C*, cultures of primary hippocampal neurons (DIV 10–14) from tau-deficient (*Mapt*^−/−^) or PrP^C^-deficient (*Prnp*^−/−^) mice were treated with Aβ oligomers (2 μm) or vehicle (*Veh*; 0.08% DMSO) for 2 h followed by Western blot analysis of sGluN1 and tGluN1 levels. *A*, representative Western blots. The *red bar* represents the boundary between different membranes. *B* and *C*, quantitation of sGluN1 (*B*) and tGluN1 (*C*) levels. *n* = 12–14 wells per condition from three to four independent experiments. The degree of sGluN1 depletion in these cultures was comparable with that in control wild-type hippocampal cultures assessed in side-by-side experiments (data not shown). *D--G*, cultures of primary hippocampal neurons (DIV 10–14) from wild-type mice were treated with Aβ oligomers or vehicle for 2 h followed by Western blot analysis of sGluN1, tGluN1, phosphorylated (*p*) ERK, and total (*t*) ERK levels. Some cultures were treated with TTX (1 μm) 30 min before and throughout exposure to Aβ or vehicle. Phospho-ERK levels were normalized to total ERK levels. *D*, representative Western blot. *E–G*, quantitations of sGluN1 (*E*), tGluN1 (*F*), and phospho-ERK (*G*) levels. For ERK levels, the sum of two bands (ERK1 and -2) was quantitated. *n* = 16 wells per condition from four independent experiments. Two-way ANOVA revealed an interaction between the effects of Aβ and TTX on phospho-ERK levels (*p* < 0.05) but not on sGluN1 levels (*p* = 0.98). One-way ANOVA revealed a significant (*p* < 0.001) TTX effect on phospho-ERK levels. *, *p* < 0.05; **, *p* < 0.01 *versus* vehicle by unpaired *t* test with Welch's correction (*B*) or Bonferroni test (*E*). *Bars* and *error bars* represent means and S.E., respectively.

**FIGURE 5.**
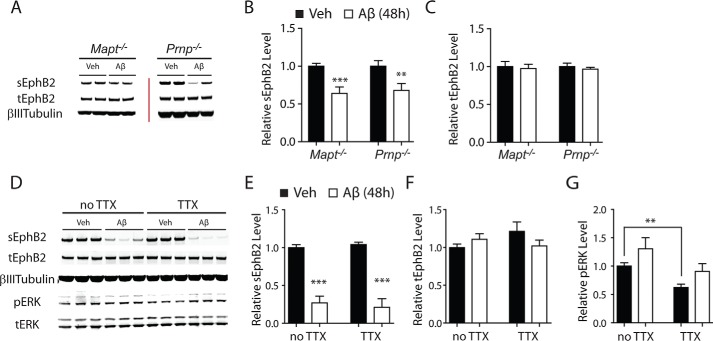
**Aβ-induced depletion of sEphB2 occurs in the absence of tau, PrP^C^, or neuronal activity.** *A–C*, cultures of primary hippocampal neurons (DIV 10–14) from tau-deficient (*Mapt*^−/−^) or PrP^C^-deficient (*Prnp*^−/−^) mice were treated with Aβ oligomers (2 μm) or vehicle (*Veh*; 0.08% DMSO) for 48 h followed by Western blot analysis of sEphB2 and tEphB2 levels. *A*, representative Western blots. The *red bar* represents the boundary between different membranes. *B* and *C*, quantitation of sEphB2 (*B*) and tEphB2 (*C*) levels. *n* = 12–18 wells per condition from three to four independent experiments. *D--G*, cultures of primary hippocampal neurons (DIV 10–14) from wild-type mice were treated with Aβ oligomers or vehicle for 48 h followed by Western blot analysis of sEphB2, tEphB2, phosphorylated (*p*) ERK and total (*t*) ERK levels. Some cultures were treated with TTX (1 μm) from 30 min before until the end of exposure to Aβ or vehicle. *D*, representative Western blot. *E–G*, quantitations of sGluN1 (*E*), tGluN1 (*F*), and phospho-ERK (*G*) levels. *n* = 12 wells per condition from three independent experiments. **, *p* < 0.01; ***, *p* < 0.001 *versus* vehicle by unpaired *t* test with Welch's correction (*B*) or Bonferroni test (*E* and *G*). *Bars* and *error bars* represent means and S.E., respectively.

We next investigated whether potential mediators of Aβ-induced neuronal impairments, specifically tau, PrP^C^, and aberrant excitatory neuronal activity (15–17, 19–21, 23–27), are required for Aβ-induced depletion of sGluN1 and sEphB2. Treatment with Aβ oligomers depleted sGluN1 (Fig. 4, *A–F*) and sEphB2 (Fig. 5, *A–F*) in primary hippocampal neurons from mice lacking tau or PrP^C^ and in wild-type neurons treated with TTX, which blocks action potentials by inhibiting voltage-gated sodium channels. The extent of Aβ-induced sGluN1 and sEphB2 depletion in PrP^C^-deficient (Figs. 4, *A* and *B*, and 5, *A* and *B*), tau-deficient (Figs. 4, *A* and *B*, and 5, *A* and *B*), and TTX-treated wild-type (Figs. 4, *D* and *E*, and 5, *D* and *E*) cultures was comparable with that in wild-type control cultures (Fig. 1, *A* and *B*). To confirm that TTX was effective in these experiments, we measured levels of phosphorylated (*i.e.* activated) ERK, which TTX reduced (Figs. 4*G* and 5*G*). Thus, tau, PrP^C^, and aberrant excitatory neuronal activity are unlikely mediators of the Aβ-induced depletion of sGluN1 and sEphB2, at least in these culture models.

##### Ability of EphB2 to Prevent Aβ-induced Depletion of sGluN1 Depends on Its PDZ-binding Motif

**FIGURE 6.**
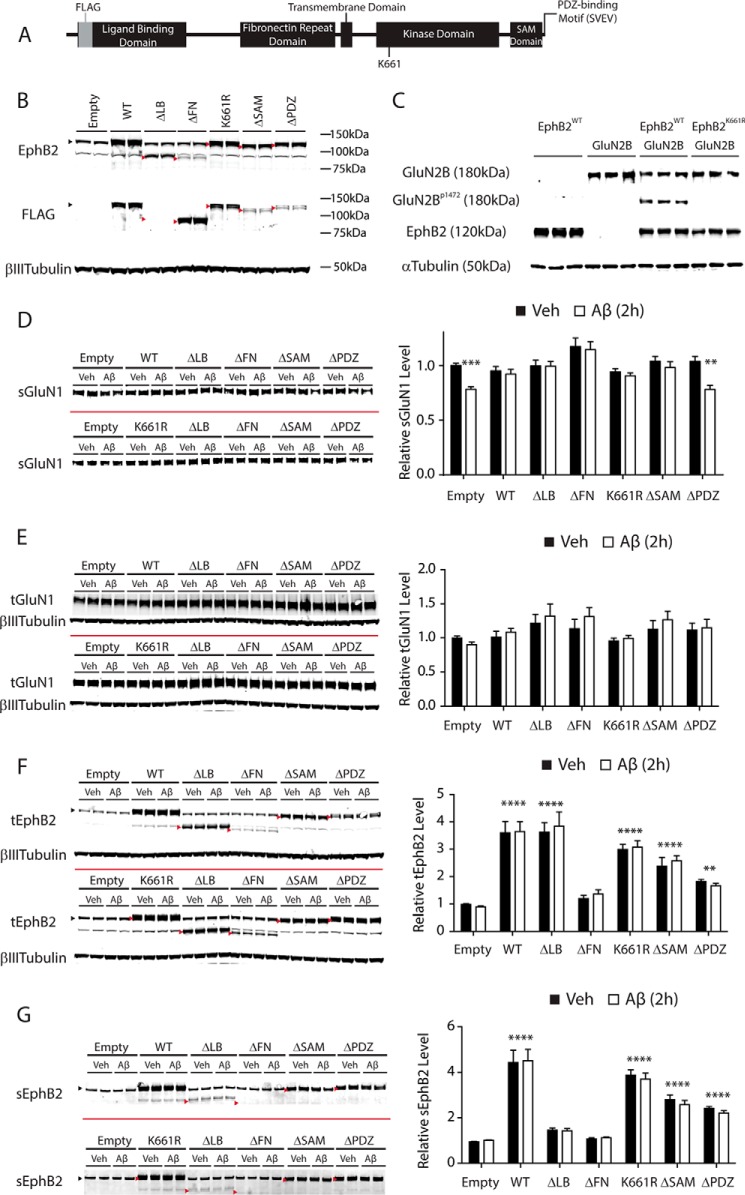
**Prevention of Aβ-induced GluN1 depletion by overexpression of EphB2 depends on PDZ-binding motif of EphB2.** *A*, schematic diagram of FLAG-tagged mouse EphB2 showing functionally important domains and amino acid residues. The last four amino acids in the C terminus (SVEV; the PDZ-binding motif) are critical for binding to PDZ domain-containing proteins. Lysine 661 (*K661*) is critical for kinase activity of EphB2. *B*, expression of different EphB2 constructs in primary neurons. Cultures of hippocampal neurons (DIV 2) from wild-type mice were transduced (0.02 pg of p24/neuron) with empty lentivirus (*Empty*) or with lentivirus encoding EphB2^WT^ (*WT*), EphB2^ΔLB^ (Δ*LB*), EphB2^ΔFN^ (Δ*FN*), EphB2^K661R^ (*K661R*), EphB2^ΔSAM^ (Δ*SAM*), or EphB2^ΔPDZ^ (Δ*PDZ*) followed by Western blot analysis for EphB2 and FLAG on DIV 14. Except for EphB2^ΔLB^, all constructs contained a FLAG tag at the N-terminal side of the ligand-binding domain (*A*). The same Western blot was probed with a polyclonal anti-EphB2 antibody (*top*) and a monoclonal anti-FLAG antibody (*middle*) and labeled with secondary antibodies conjugated to distinct fluorophores. Wild-type and mutant EphB2 bands are indicated by *black* and *red arrowheads*, respectively. The faint bands between 75 and 100 kDa in the EphB2 blot are probably nonspecific as they were also present in primary neurons from EphB2-deficient mice (data not shown). *C*, substitution of Lys-661 for arginine (K661R) specifically disrupts the kinase activity of EphB2. HEK293T cells were transiently transfected with plasmids encoding EphB2^WT^ or GluN2B or co-transfected with GluN2B plus EphB2^WT^ or EphB2^K661R^. Two days after the transfection, cells were treated for 1 h at 37 °C with preclustered human Fc-EphrinB2 (500 ng/ml) followed by Western blot analysis for GluN2B, GluN2B phosphorylated at tyrosine 1472 (*GluN2B^p1472^*), EphB2, and α-tubulin. *D--G*, cultures of primary hippocampal neurons (DIV 2) from wild-type mice were transduced with different EphB2 constructs as in *B*. At DIV 12–14, neurons were treated with Aβ oligomers (2 μm) or vehicle (*Veh*; 0.08% DMSO) for 2 h followed by quantification of sGluN1 (*D*), tGluN1 (*E*), tEphB2 (*F*), and sEphB2 (*G*) levels by Western blot analysis. Representative Western blots are shown on the *left*, and quantitations of Western blot signals are shown on the *right*. The *red bars* represent the boundary between different membranes. Two representative Western blots with duplicate samples for some EphB2 constructs are shown. *n* = 15–33 wells per condition from four to nine independent experiments. In *D*, overexpression of each EphB2 construct, except for EphB2^ΔPDZ^, prevented the Aβ-induced depletion of sGluN1. In *E* and *F*, the βIII-tubulin blots are the same. In *F* and *G*, bands of EphB2^WT^, EphB2^K661R^, EphB2^ΔSAM^, and EphB2^ΔPDZ^ overlap with that of endogenous EphB2 at ∼120 kDa, whereas bands of EphB2^ΔLB^ and EphB2^ΔFN^ reside below the faint nonspecific band around 80 kDa (see also *B*). Wild-type and mutant EphB2 bands are indicated by *black* and *red arrowheads*, respectively. Quantitations of EphB2 levels represent the sum of endogenous (∼120 kDa) and exogenous (WT or mutant) EphB2 signals. Two-way ANOVA revealed a significant (*p* < 0.01) interaction between the effects of Aβ treatment and EphB2 transduction on sGluN1 levels (*D*), significant effects of EphB2 constructs (*p* < 0.0001) but not of Aβ, and no significant interaction between EphB2 constructs and Aβ (*F* and *G*). **, *p* < 0.01; ***, *p* < 0.001; ****, *p* < 0.0001 *versus* vehicle condition (*D*) or empty condition (*F* and *G*) by Bonferroni test. *Bars* and *error bars* represent means and S.E., respectively.

We previously showed that normalizing EphB2 levels in the dentate gyrus of hAPP transgenic mice reversed deficits in NMDAR function (32), and others recently showed that EphB2 overexpression also counteracts Aβ-induced NMDAR dysfunctions in neuronal cultures (43). Overexpression of FLAG-tagged wild-type EphB2 (EphB2^WT^) also prevented Aβ-induced depletion of sGluN1 in neuronal cultures in the current study (Fig. 6). In one experiment, increasing the expression of EphB2^WT^ in primary cultures of rat hippocampal neurons also partially counteracted the depletion of sGluN1 caused by synthetic Aβ oligomers, which, by Western blot analysis, contained more putative higher order assemblies than recombinant Aβ oligomers (data not shown).

To begin to explore the molecular mechanism by which EphB2 exerts this protective effect, we generated multiple constructs encoding mutant forms of FLAG-tagged EphB2 (Fig. 6, *A–C*), including EphB2 lacking the ligand-binding domain (EphB2^ΔLB^), the fibronectin repeats (EphB2^ΔFN^), the SAM domain (EphB2^ΔSAM^), the PDZ-binding motif (EphB2^ΔPDZ^), or kinase activity (EphB2^K661R^). Western blot analyses of wild-type neuronal cultures transduced with the different EphB2 constructs confirmed the expected changes in molecular weight and interaction with the anti-FLAG antibody (Fig. 6*B*). Although the EphB2 antibody used in this study is polyclonal and recognized all EphB2 mutants we generated, it showed relatively less reactivity with EphB2^ΔFN^, which was recognized readily by the anti-FLAG antibody (Fig. 6*B*). Kinase-deficient EphB2^K661R^ in which the lysine 661 residue critical for tyrosine kinase activity was mutated to arginine failed to mediate ephrinB2-induced phosphorylation of the tyrosine 1472 residue (Tyr-1472) on GluN2B (Fig. 6*C*), confirming the desired impact of the mutation.

Except for EphB2^ΔPDZ^, all EphB2 mutants and EphB2^WT^ prevented Aβ-induced sGluN1 depletion when overexpressed in primary cultures of hippocampal neurons (Fig. 6*D*). As documented in Figs. 1*C* and 6*D* (*Empty*), the endogenous EphB2, which was coexpressed in these cultures, would not be expected to modulate the Aβ-induced depletion of sGluN1. It is also worth noting that deletion of the PDZ-binding motif did not alter cell surface expression, localization in spines, and ephrin-dependent clustering of FLAG-tagged EphB2 (44), suggesting that the lack of protective effect by EphB2^ΔPDZ^ was not caused by alterations in its subcellular localization.

Over the time frame of this experiment, levels of total GluN1 were not significantly altered by treatment with Aβ oligomers or expression of EphB2 (Fig. 6*E*). The degree of EphB2 overexpression we achieved by lentiviral transduction of neurons was variable across constructs as determined by Western blot analysis of neuronal lysates (Fig. 6, *F* and *G*), possibly reflecting differences in the reactivity of EphB2 mutants with the anti-EphB2 antibody or in their stability. For most constructs, levels of sEphB2 correlated well with those of total EphB2 (tEphB2); however, sEphB2^ΔLB^ levels were low despite high levels of tEphB2^ΔLB^ expression (Fig. 6, *F* and *G*). Surface levels of EphB2^ΔLB^ and likely EphB2^ΔFN^ may have appeared lower than they actually were because these constructs lack large portions of the extracellular part of the molecule (Fig. 6*A*) and thus might have been less efficiently biotinylated and/or pulled down. Regardless, these two EphB2 mutants were able to counteract Aβ-induced sGluN1 depletion.

**FIGURE 7.**
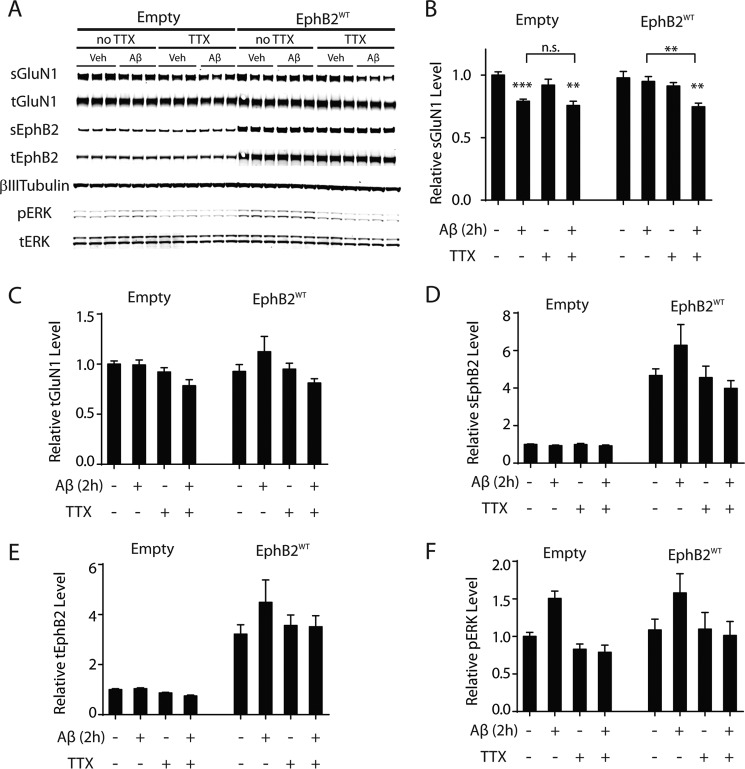
**Prevention of Aβ-induced GluN1 depletion by overexpression of EphB2 depends on neuronal activity.** Silencing neuronal activity by treating neurons with TTX (1 μm) for 30 min before and throughout the Aβ exposure prevented lentivirus-mediated overexpression of EphB2^WT^ from blocking the Aβ-induced depletion of sGluN1. Cultures of primary hippocampal neurons (DIV 2) from wild-type mice were transduced (0.02 pg of p24/neuron) with empty lentivirus (*Empty*) or with lentivirus encoding EphB2^WT^. At DIV 12–14, cultures were treated with Aβ oligomers (2 μm) or vehicle (*Veh*; 0.08% DMSO) for 2 h followed by Western blot analysis. Cultures were or were not treated with TTX (1 μm) 30 min before and throughout Aβ or vehicle treatment as indicated. Treatments are shown below the bar graphs; whether Aβ oligomers or TTX (+) or vehicle (−) was applied is indicated. *n* = 9–10 wells per condition from three independent experiments. *A*, representative Western blot. *tERK*, total ERK. *B–F*, quantitation of Western blot signals for sGluN1 (*B*), tGluN1 (*C*), sEphB2 (*D*), tEphB2 (*E*), and phosphorylated (*p*) ERK (*F*). In *B*, two-way ANOVA revealed no significant interaction between the effects of Aβ and TTX on sGluN1 levels in cultures transduced with empty (*p* = 0.49) or EphB2^WT^ (*p* = 0.09)-expressing lentivirus. For the empty condition in *F*, two-way ANOVA revealed a significant effect of TTX (*p* < 0.001) and of Aβ (*p* < 0.01) and significant (*p* < 0.01) interaction. *n.s.*, not significantly different; **, *p* < 0.01; ***, *p* < 0.001 *versus* vehicle condition or as indicated by *brackets* (Bonferroni test). *Bars* and *error bars* represent means and S.E., respectively.

Notably, overexpressing EphB2 did not prevent Aβ-induced GluN1 depletion when neuronal cultures were treated with TTX (Fig. 7, *A* and *B*), suggesting that neuronal activity is required for EphB2 overexpression to counteract this Aβ effect. Treatment of cultures with Aβ oligomers or TTX and overexpression of EphB2^WT^ did not significantly alter total GluN1 levels (Fig. 7, *A* and *C*). Independently of whether cultures were treated with Aβ oligomers, TTX, or both, overexpression of EphB2^WT^ increased levels of total EphB2 and sEphB2 roughly 4–6-fold over endogenous EphB2 levels found in cultures transduced with control virus (Fig. 7, *A*, *D*, and *E*). We again confirmed the efficacy of TTX in this experiment by monitoring phospho-ERK levels (Fig. 7, *A* and *F*).

##### Ability of EphB2 to Counteract the Effect of Aβ Oligomers May Depend on Its Interaction with GluA2

EphB2 can phosphorylate NMDARs through its tyrosine kinase activity and can directly bind to them via its extracellular domains (33). However, overexpression of EphB2 mutants lacking kinase activity or specific extracellular domains still prevented Aβ-induced depletion of surface GluN1, which makes it unlikely that the protective ability of EphB2 is mediated by direct effects on NMDARs. Therefore, we focused on GluA2, which can become indirectly associated with the PDZ-binding motif of EphB2 through direct interactions with PDZ domain-containing proteins (44, 45). Additionally, endocytosis of GluA2 is required for Aβ to depress NMDAR currents and synaptic function (9).

**FIGURE 8.**
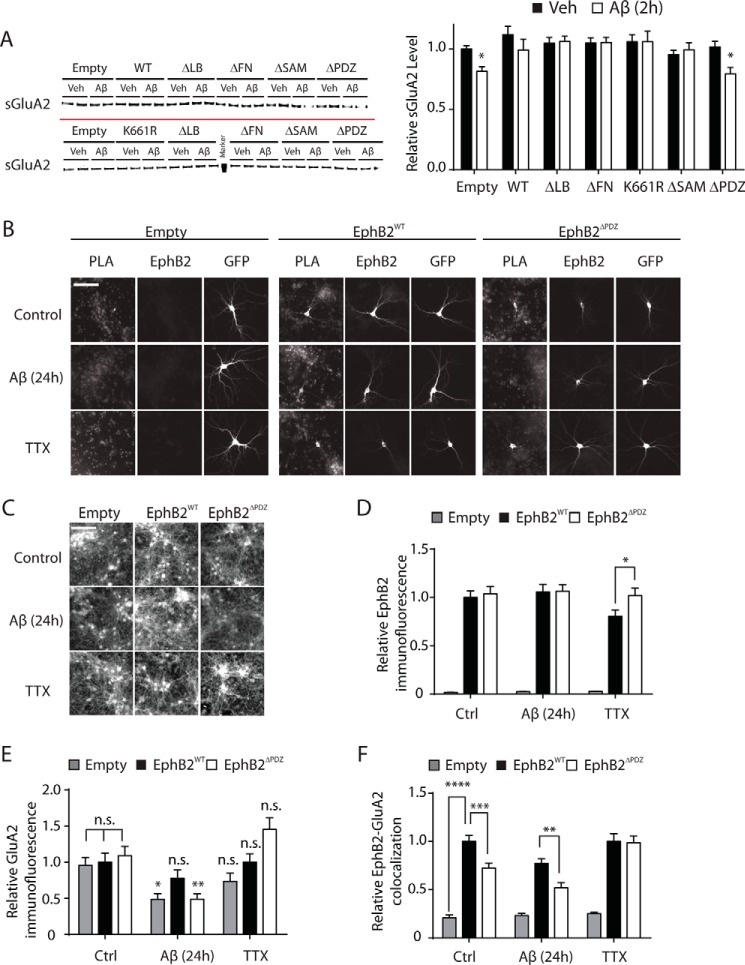
**Prevention of Aβ-induced GluA2 depletion by overexpression of EphB2 also depends on PDZ-binding motif of EphB2 and may involve interactions between GluA2 and EphB2.** *A*, overexpression of EphB2 prevents Aβ-induced depletion of sGluA2, and this effect requires the PDZ-binding motif of EphB2. Neurons were transduced with different EphB2 constructs and treated with Aβ as described in Fig. 6, *D–G*, followed by quantitation of sGluA2 levels by Western blot analysis. Two representative Western blots are shown on the *left*, and quantitations of Western blot signals are shown on the *right. n* = 15–18 wells per condition from three to four independent experiments. Two-way ANOVA revealed a significant (*p* < 0.05) interaction between the effects of Aβ treatment and EphB2 transduction on sGluA2 levels. *B–F*, putative interactions between EphB2 and GluA2 in individual transfected neurons were monitored with a PLA in which fluorescence signal above background indicates close proximity (<40 nm) between EphB2 and GluA2. Cultures of primary hippocampal neurons (DIV 7) from wild-type mice were transfected with empty pFUW plasmid (*Empty*) or with pFUW plasmid encoding EphB2^WT^ or EphB2^ΔPDZ^. GFP-encoding plasmid was co-transfected to visualize transfected neurons. Some cultures were treated with Aβ oligomers (2 μm) or vehicle (*Veh*; 0.08% DMSO) for 24 h, and others were treated with TTX (1 μm) or vehicle for 6 h prior to fixation. Fixed cultures were analyzed by PLA and immunostained for EphB2 or GluA2. *B*, for each treatment and transfection condition, PLA signals are shown on the *left*, EphB2 immunoreactivity is shown in the *middle*, and GFP fluorescence is shown on the *right*. GFP fluorescence was observed only in transfected neurons. EphB2 images were also thresholded to identify primarily EphB2-transfected neurons, which displayed stronger EphB2 immunoreactivity than untransfected neurons expressing only endogenous EphB2. *Scale bar*, 100 μm. *C*, GluA2 immunostaining for each treatment and transfection condition. *Scale bar*, 100 μm. *D* and *E*, relative intensity of EphB2 (*D*) and GluA2 (*E*) immunofluorescence in cell bodies of GFP-positive neurons. *n* = 16–59 neurons per condition from two independent experiments. *F*, relative PLA signal indicating EphB2-GluA2 colocalization. EphB2-GluA2 interactions were 1) increased when EphB2 was overexpressed, 2) reduced by Aβ, and 3) partly dependent on the PDZ-binding motif of EphB2. *n* = 51–85 neurons per condition from four independent experiments. Two-way ANOVA revealed significant effects of EphB2 constructs (*p* < 0.0001), Aβ (*p* < 0.05), and TTX (*p* < 0.05) and no significant interactions between EphB2 constructs and Aβ or TTX. *n.s.*, not significantly different; *, *p* < 0.05; **, *p* < 0.01; ***, *p* < 0.001; ****, *p* < 0.0001 *versus* vehicle (*A*) or *versus* control (*Ctrl*) condition or as indicated by *brackets* (*D–F*) by Bonferroni test. *Bars* and *error bars* represent means and S.E., respectively.

Treatment of neuronal cultures with Aβ oligomers decreased surface levels of the AMPAR subunit GluA2 (Fig. 8*A*), consistent with previous reports (9, 13, 14). sGluA2 was depleted by Aβ within 2 h, which is similar in time frame to the depletion of sGluN1 and much faster than the depletion of sEphB2. Interestingly, EphB2 overexpression counteracted the Aβ-induced depletion of sGluA2 through a mechanism that depended on the presence of the PDZ-binding motif of EphB2 (Fig. 8*A*). To monitor interactions between EphB2 and GluA2 in primary neuronal cultures, we used a PLA that allows for the *in situ* detection of two antigens only when they are in close proximity (<40 nm) (38).

At comparable levels of overexpression, EphB2^WT^ showed more colocalization with GluA2 than EphB2^ΔPDZ^ independently of whether cultures were treated with vehicle or Aβ (Fig. 8, *B–F*). Treatment with Aβ oligomers reduced the colocalization of EphB2 and GluA2 in neurons overexpressing EphB2^WT^ or EphB2^ΔPDZ^ (*p* < 0.01 by two-way ANOVA). TTX treatment had no effect on colocalization between GluA2 and endogenous EphB2 or overexpressed EphB2^WT^ but increased colocalization between GluA2 and overexpressed EphB2^ΔPDZ^ (Fig. 8*F*), possibly due to increased surface GluA2 resulting from synaptic scaling (46). For unclear reasons, TTX treatment reduced the levels of overexpressed EphB2^WT^ but not EphB2^ΔPDZ^ (Fig. 8*D*). This difference may have obscured some effects of the mutation, for example, resulting in the detection of comparable levels of interaction between GluA2 and EphB2^WT^ *versus* EphB2^ΔPDZ^ in the presence of TTX (Fig. 8*F*). GluA2 levels were not altered by overexpression of EphB2^WT^ or EphB2^ΔPDZ^ under control conditions (Fig. 8*E*). GluA2 levels were reduced by Aβ in neurons that were transfected with empty vector or vector encoding EphB2^ΔPDZ^ but not in those overexpressing EphB2^WT^ (Fig. 8*E*). Taken together, these results suggest that EphB2 overexpression may counteract Aβ-induced depletion of sGluN1 by increasing sGluA2 levels.

**FIGURE 9.**
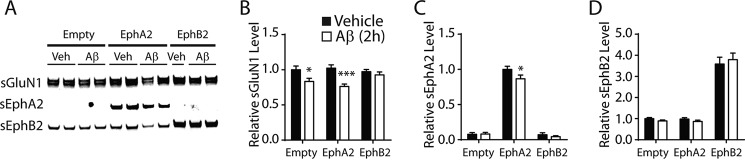
**Overexpression of EphA2, another receptor tyrosine kinase with a PDZ-binding motif, does not prevent Aβ-induced GluN1 depletion.** *A–D*, cultures of primary hippocampal neurons (DIV 2) from wild-type mice were transduced with empty lentivirus (*Empty*) or lentivirus encoding EphA2 or EphB2. At DIV 14, neurons were treated with Aβ oligomers (2 μm) or vehicle (*Veh*; 0.08% DMSO) for 2 h followed by Western blot analysis. *A*, representative Western blots. *B–D*, quantification of sGluN1 (*B*), sEphA2 (*C*), and sEphB2 (*D*) signals. *n* = 7–8 wells per condition from two independent experiments. In *C*, two-way ANOVA revealed a significant (*p* < 0.0001) effect of Aβ but not of lentivirus and no significant interaction between them. *, *p* < 0.05; ***, *p* < 0.001 *versus* vehicle by Bonferroni test. *Bars* and *error bars* represent means and S.E., respectively.

Lastly, we tested and refuted the hypothesis that overexpression of any protein bearing a PDZ-binding motif can counteract the effect of Aβ oligomers on cell surface glutamate receptors. For this purpose, we focused on EphA2, another receptor tyrosine kinase with a PDZ-binding motif (47). To our knowledge, EphA2 has not been demonstrated to interact with or regulate glutamate receptors. Overexpression of EphA2 in cultured neurons did not prevent Aβ-induced depletion of sGluN1 (Fig. 9).

## Discussion

This study demonstrates that the ability of EphB2 to counteract Aβ-induced depletions of AMPARs and NMDARs depends on its PDZ-binding motif and the presence of neuronal activity. We also obtained evidence suggesting that this effect may involve interactions between the PDZ-binding motif of EphB2 and GluA2, which could promote the retention of GluA2 at the surface membrane and prevent Aβ-induced depletion of surface NMDARs (9, 14). From a therapeutic perspective, it is important to note that these protective EphB2 effects were observed only when EphB2 was expressed at supraphysiological levels and that they did not depend on its kinase activity.

We also found that Aβ-induced depletions of NMDARs do not depend on EphB2 depletion, tau, PrP^C^, or aberrant neuronal activity, all of which have been implicated in Aβ-induced neuronal dysfunction (12, 15–17, 20, 21, 23–27, 31, 32, 43). One interpretation of these findings is that NMDAR depletion occurs upstream of the other factors within the pathogenic cascade that Aβ oligomers trigger. However, we found that Aβ oligomers depleted EphB2 even in the presence of cyclosporin or AP5, which prevented Aβ-induced depletion of NMDARs. An alternative possibility is that Aβ oligomers activate parallel pathways that affect neuronal functions through distinct mechanisms.

It should be noted in this context that all our findings were obtained in dissociated primary neuronal cultures. We cannot exclude the possibility that the relationships among the factors we studied are different *in vivo*. As is true for most data obtained in experimental models, the relevance of our findings to patients with AD also remains uncertain and deserves to be further explored in future studies. Notwithstanding these caveats, the novel mechanistic insights our study provides could guide the development of strategies to counteract Aβ-induced neuronal dysfunction and help make the brain more resistant against pathogenic Aβ assemblies.

Aβ oligomers deplete and dysregulate glutamate receptors and related molecules, including EphB2 (7, 9–12, 14, 32). However, it has remained uncertain whether these alterations are causally linked with each other and, if so, in which sequence or constellation. Our results suggest that Aβ-induced endocytosis of GluA2 acts upstream of and may promote the depletion of GluN1, consistent with previous studies showing that GluA2 endocytosis is required for Aβ-induced NMDAR dysfunction and synaptic depression (9, 14). Interestingly, the Aβ-induced depletions of GluA2 and GluN1 could be prevented by neuronal overexpression of EphB2^WT^ but not EphB2^ΔPDZ^. EphB2 regulates trafficking of GluA2 by a mechanism that depends on its PDZ-binding motif: the PDZ-binding motif of EphB2 binds to the PDZ domain-containing scaffold protein glutamate receptor-interacting protein 1, which binds to GluA2 and regulates the localization of GluA2-containing AMPARs (33, 44, 45). Using a PLA approach, we confirmed that this motif is required for the association of EphB2 with GluA2.

In contrast, the PDZ-binding motif does not appear to be involved in interactions between EphB2 and NMDARs. Instead, EphB2 can regulate NMDAR localization and function by directly binding to GluN1 through its extracellular domain and phosphorylating GluN2B through its kinase activity (33, 39, 48). However, although the kinase activity of EphB2 can influence NMDAR localization and function *in vitro* (39), NMDAR impairments in EphB2-deficient mice could be normalized by expression of kinase-deficient EphB2 (49), suggesting a more limited or different role for this activity *in vivo*.

In the current study, the prevention of Aβ-induced GluN1 depletion by overexpression of EphB2 was independent of the kinase activity of EphB2 as well as of its ligand-binding domain and fibronectin repeats, which together make up most of the extracellular domain. In our view, the most parsimonious interpretation of these findings is that overexpression of EphB2 prevents Aβ-induced NMDAR depletion by PDZ-binding motif-mediated retention of GluA2 at the surface membrane, which would be expected to counteract Aβ-induced enhancement of GluA2 endocytosis and consequent GluN1 depletion (9).

Because this protective effect of EphB2 was observed only when it was overexpressed and Aβ oligomers depleted sGluN1 to similar degrees in wild-type and EphB2-deficient neurons, it is likely that the association between endogenous EphB2 and GluA2 is not strong enough to retain GluA2 at the surface, at least in the presence of pathologically elevated levels of Aβ oligomers. However, because genetic ablation of EphB2 during early stages of development may engage compensatory mechanisms (32, 50), it remains possible that endogenous EphB2 counteracts the Aβ-induced depletion of sGluN1 in wild-type neurons of the adult brain.

It is interesting that neuronal activity was required for overexpression of EphB2 to prevent Aβ-induced GluN1 depletion even though neuronal activity did not influence the association between EphB2 and GluA2. These results suggest that neuronal activity plays a role downstream of GluA2 engagement, possibly through homeostatic synaptic scaling (46, 51).

In another potential pathway, Aβ oligomers were proposed to serially activate α-7 and striatal enriched protein tyrosine phosphatase (STEP_61_), leading to dephosphorylation of GluN2B by STEP_61_ and depletion of GluN1 (7). In contrast to results obtained by Snyder *et al.* (7), the α-7 antagonist BTX did not prevent Aβ-induced GluN1 depletion in the current study. In both studies, high concentrations (10 μm) of BTX were used as compared with its subnanomolar IC_50_ (52). Therefore, variable degrees of α-7 blockade are unlikely to account for the discrepancy. In a similar vein, although the calcineurin inhibitors FK506 and cyclosporin A have been reported to prevent APP/Aβ-induced NMDAR dysfunction (7, 9), FK506 was ineffective in our study, and cyclosporin A prevented Aβ-induced GluN1 depletion during the first 2 h as reported previously (7) but not after 48 h of Aβ exposure. Differential effects of these calcineurin inhibitors (53) and differences in experimental systems (organotypic slice culture *versus* primary neuronal culture and APP overexpression *versus* exposure to recombinant Aβ(1–42) oligomers) may explain the different results. Notably, both genetic ablation (10) and pharmacological blockade (7) of α-7 blocked Aβ-induced GluN1 depletion only partially. Based on our results and those obtained by others (9), we suspect that enhanced GluA2 endocytosis contributes to the Aβ-induced depletion of NMDARs more strongly than activation of α-7 and STEP_61_.

Although exposure to Aβ oligomers depleted both sEphB2 and sGluN1 and the Aβ-induced depletion of GluN1 could be prevented by overexpression of EphB2, the depletion of GluN1 did not depend on the depletion of EphB2, suggesting that Aβ oligomers deplete EphB2 and GluN1 through independent mechanisms. Notably, the Aβ oligomers generated from recombinant Aβ(1–42) in the current study depleted surface, but not total, EphB2 even when we used them at high concentration (up to 5 μm) or exposed cultures to them for longer periods (up to 6 days) (data not shown). These results are consistent with those obtained in a previous study using similar Aβ oligomers (12) but differ from those we obtained with naturally secreted Aβ oligomers from 7PA2-CHO cells that depleted both surface and total EphB2 (32). The discrepancy could be due to differences in the Aβ preparations used. Although increasing the expression of EphB2 counteracted the depletion of sGluN1 by recombinant or synthetic Aβ oligomers that differed in their content of putative higher order assemblies, it remains possible that overexpression of EphB2 protects against the detrimental effects of some types of Aβ oligomers but not others.

Our findings that Aβ oligomers depleted both NMDARs and AMPARs in neuronal cultures are consistent with those of previous studies (7, 9, 10, 12, 14, 32, 54, 55, 57). We also demonstrated that overexpression of EphB2 rescued both types of glutamate receptors in these culture models. In contrast, electrophysiological recordings in acute hippocampal slices from hAPP-J20 mice have so far revealed primarily deficits in the function of NMDARs but not of AMPARs (32, 57), and normalization of neuronal EphB2 levels in the dentate gyrus of these mice appeared to specifically rescue NMDAR function (32). These discrepancies may be due to differences in the (*a*) duration of exposure to elevated Aβ levels (several months in hAPP-J20 mice *versus* 2 or 48 h in the current study), (*b*) APP metabolites (mixture of Aβ peptides and other APP metabolites produced by neurons in brain *versus* recombinant Aβ(1–42) added to cell culture medium), (*c*) cell types exposed to Aβ (diverse populations of mature neurons, glia, and endothelial cells in hAPP-J20 mice *versus* primary cultures enriched for young hippocampal neurons), and (*d*) duration and extent of EphB2 expression (several months of normalized levels in hAPP-J20 mice *versus* 10–12 days of overexpression in cultured hippocampal neurons). Thus, the mechanisms by which EphB2 normalization reversed functional deficits in hAPP-J20 mice may well have been at least partly different from those by which EphB2 overexpression prevented depletion of surface glutamate receptors in the current study.

Although many Aβ-induced effects on neuronal integrity and function depend on the presence of tau (15–17, 58–66), others do not. In the current study, tau was not required for Aβ oligomers to deplete GluN1 and EphB2. Tau also does not appear to be required for the Aβ-induced loss of dendritic spines (60, 67). Given the roles of GluN1 and EphB2 in the formation and maintenance of dendritic spines (33, 68), it is tempting to speculate that the depletion of these molecules promotes spine loss.

Another factor that has been implicated in Aβ-induced neuronal dysfunction is PrP^C^. However, different groups have obtained perplexingly disparate results in regard to this potential mediator. Indeed, PrP^C^ has been shown to be required for hAPP/Aβ to impair neuronal functions *in vitro* (24, 25, 69–71) and *in vivo* (27, 71). In contrast, other groups have identified various hAPP/Aβ-induced neuronal impairments that do not depend on the presence of PrP^C^ (28–30, 71, 72). In the current study, PrP^C^ ablation did not prevent Aβ-induced sGluN1 depletion, contrary to results obtained by Um *et al.* (25). Although differences between the specific Aβ assemblies used in these studies may explain the discrepancy (56), our findings clearly demonstrate that some Aβ oligomers can deplete surface NMDARs independently of any mediation by PrP^C^. Conceivably, different types of Aβ oligomers cause neuronal dysfunctions by engaging different mediators. Further adding to this complexity, our findings suggest that even the same type of Aβ oligomers can impair neuronal functions through distinct pathways that, at a minimum, involve GluA2/GluN1 and EphB2.

## Author Contributions

T. M. designed, conducted, and analyzed all the experiments and wrote the manuscript. D. K. characterized Aβ oligomers by AFM. J. A. K. contributed to the proximity ligation assay shown in Fig. 8, *B–F*. E. J. contributed to the characterization of Aβ oligomers. L. M. supervised the study and wrote the manuscript. All authors reviewed the results and approved the final version of the manuscript.

## Footnotes

3 The abbreviations used are:

AD: Alzheimer disease
Aβ: amyloid-β
NMDAR: NMDA-type glutamate receptor
AMPAR: AMPA-type glutamate receptor
PrP^C^: prion protein
(h)APP: (human) amyloid precursor protein
DMSO: dimethyl sulfoxide
AFM: atomic force microscopy
DPBS: Dulbecco's phosphate-buffered saline
SEC: size exclusion chromatography
DIV: day(s) *in vitro*
Bis-Tris: 2-[bis(2-hydroxyethyl)amino]-2-(hydroxymethyl)propane-1,3-diol
Tricine: *N*-[2-hydroxy-1,1-bis(hydroxymethyl)ethyl]glycine
VSVG: vesicular stomatitis virus G
PLA: proximity ligation assay
TTX: tetrodotoxin
ANOVA: analysis of variance
sGluN1: surface GluN1
sEphB2: surface EphB2
BTX: α-bungarotoxin
α-7: α-7 nicotinic acetylcholine receptor(s)
AP5: d-2-amino-5-phosphonopentanoate
SAM: sterile α motif
sGluA2: surface GluA2
tEphB2: total EphB2
tGluN1: total GluN1.
